# Iontophoresis as an Effective Drug Delivery System in Dentistry: A Review

**DOI:** 10.7759/cureus.30658

**Published:** 2022-10-25

**Authors:** Rutuja Ragit, Punit Fulzele, Nilesh V Rathi, Nilima R Thosar, Monika Khubchandani, Nishi S Malviya, Simran Das

**Affiliations:** 1 Pediatric and Preventive Dentistry, Sharad Pawar Dental College and Hospital, Datta Meghe Institute of Medical Sciences (Deemed to be University), Wardha, IND; 2 Pediatric Dentistry, Sharad Pawar Dental College and Hospital, Datta Meghe Institute of Medical Sciences (Deemed to be University), Wardha, IND; 3 Pediatric Dentistry, Dr. D. Y. Patil Dental College and Hospital, Dr. D. Y. Patil Vidyapeeth, Pune, IND

**Keywords:** nano-materials, remineralization, hypersensitivity, incipient caries, iontophoresis

## Abstract

Iontophoresis is a non-invasive method to improve drug delivery by the application of an electric field. The iontophoresis process causes deeper penetration of ions using electric current. The drug delivered through iontophoresis was found to be around 10 to 2,000 times more than conventional forms of delivery. The better results were shown by alternating current (AC) than conventional constant current (DC) iontophoresis. The preparation used in iontophoresis should be soluble in water, of a small voltage, and prone to ionization. More mobility is seen with smaller particles. Iontophoresis could increase the diffusion of drugs into dentin, enamel, and other oral tissues. The chief drugs delivered or studied by iontophoresis in dentistry are non-steroidal anti-inflammatory drugs, local anesthetics, anti-bacterial drugs, and fluorides. To enhance the ability of drug transfer nanomaterials were introduced. Under the impact of iontophoresis, remineralizing nanomaterial can be injected at larger concentrations in the deeper layer of incipient caries. Due to the size of nanocomplexes, it is possible that they will diffuse into the body of the subsurface lesion and enter the porosities to improve remineralization utilizing the iontophoresis approach. The concept of the application of an electric current for drug delivery was introduced several years ago in clinical practice, research, and literature. This review focuses on iontophoresis application in dentistry, its mode of action, and how the technique can be utilized in a beneficial way.

## Introduction and background

The most mineralized structure is enamel, which includes 96% minerals. However, it also has some organic matrix (0.6%) and water (3.4%) in two states, including 1% free water. As a result, some ions and molecules diffuse through the enamel structure. The initial carious lesion is visible under the dental plaque and has a typical surface zone, a subsurface (or lesion body), a dark zone, and a translucent zone. In the surface zone of the artificial carious lesion, the microchannels are around 0.5-1.5 µm in diameter and approximately 100 µm in length [1]. Dental caries is a plaque-derived oral disease [2]. Reduced oxygen levels in deep biofilm layers make it easier for bacteria to use the glycolytic pathways to break down carbohydrates. The pH level within the biofilm is then decreased by the lactic acid produced as a byproduct, which causes subsurface demineralization [1]. To lower the morbidity, early restoration of this subsurface demineralization is advised. The non-cavitated carious lesion is successfully treated using various techniques and materials [3]. Some studies showed that the entry of restorative material into the subsurface enamel is prevented by the smaller pore size of enamel at the superficial surface [1]. Therefore, it is necessary to use materials with smaller particle sizes to enable the deposition at larger concentrations. Due to the small size of nanocomplexes, it is possible that they will diffuse into the body of the subsurface lesion and enter the porosities to promote remineralization. Nanoparticle products have shown improved phosphate and calcium precipitation in the tooth structure's deeper layer. Better methods can be used to introduce nanoparticles into the tooth structure [2]. In 1747, Pivati et al. first described iontophoresis [4]. Galvani et al., in the 18th century, revealed that electricity results in the movement of metal ions. In the early 1960s, iontophoresis was first used to treat dentin hypersensitivity [5]. Drugs were incorporated into the tissues using low-ampere electric current. It operates because opposite charges attract and like charges repel one another. It allows a concentrated form of the drug to be introduced into the needed localized area under an electrical gradient [6]. Ions undergo deeper penetration through the iontophoresis method, which uses electric current. The drug delivered through iontophoresis was around 10 to 2,000 times more than conventional delivery forms. Iontophoresis could increase the diffusion of drugs into dentin, enamel, and other oral tissues [7]. In iontophoresis, drug delivery was significantly increased compared to the passive transport of drugs [8].

## Review

Rationale

Fluoride is applied topically, causing the enamel's outer layer to remineralize without healing subsurface lesions. When an electric current is used, ions may penetrate more deeply during the iontophoresis procedure. Under the influence of iontophoresis, drugs can be administered in larger quantities in the deeper depth of tooth structure. To decrease hypersensitivity and boost remineralization, iontophoresis may be beneficial in accelerating both the rate of release and the depth of penetration of the medications.

Aim

Iontophoresis aims to intensify the beneficial effect of drugs and to infiltrate needed ions into the subsurface of enamel. This review aims to cover the utilization of an iontophoretic technique for drug transfer in the tooth structure to increase remineralization and reduce hypersensitivity.

Literature search

This review aimed to examine the literature to recognize the therapeutic use of iontophoresis and its mode of action in dentistry. Many in vivo and in vitro studies have been conducted. Various measures such as penetration depth, rate, size, and mobility of drug through iontophoresis, type and dose currently used, and its efficacy on tooth structure were considered. According to PRISMA (Preferred Reporting Items for Systematic Reviews and Meta-Analyses) guidelines, the PubMed database and cross-references were searched for literature on iontophoresis and its utilization in dentistry between 1964 and 2019. The following inclusion criteria were used: iontophoresis and its mode of action and application in dentistry, fluoride, and silver products, along with iontophoresis and its effect on the carious lesion and on tooth structure. Exclusion criteria were articles published in a language other than English.

**Figure 1:**
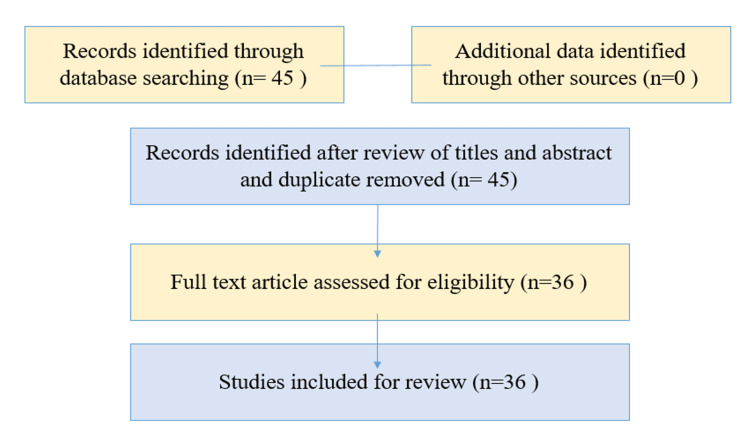
Flowchart of the literature review process

Figure 1 shows a flowchart of the literature review process.

Iontophoresis is an area that has extensive scope for growth. As this process utilizes electric current, it causes a rapid drug diffusion rate. Its mechanism of action plays an important role. Therefore, along with the mechanism of action in this review, factors affecting the iontophoretic process, drugs that are selectively used with iontophoresis, its effect on enamel and dentin surface, effects on reduction in hypersensitivity, and increasing remineralization are also described in detail.

Mechanism of iontophoresis

Delivery of uncharged and charged drugs in iontophoresis is enhanced by using electric current through a membrane. The principle of iontophoresis therapy states that ions carrying similar charges repel each other [9]. Iontophoresis works on a mechanism that involves electrophoresis, electro-osmosis, and electro-permeabilization [10-13]. In electrophoresis, the anode repels the positively charged ions, and the cathode repels negatively charged ions. This mechanism plays an important part in the increased transfer of drugs with ionic charges. Electro-osmosis is based on the passage of ionic and neutral drugs. The electric field helps transport neutral and charged molecules through a charged membrane along with the bulk solvent flow. The third mechanism is electro-permeabilization, under the impact of an electric field, which modifies the membrane barrier by increasing the intrinsic permeability and altering the permeation pathway's properties, which involve the membrane pore sizes and charges [14,15].

Factors affecting the iontophoresis

Iontophoresis is affected by many factors. Rai and Srinivas analyzed drug penetration through the skin using an external electric field, along with its properties and factors affecting it. According to this study, the drug used in iontophoresis should be soluble in water, of a small voltage, and prone to ionization. More mobility is seen with smaller particles. To some extent, there is better drug delivery when drug concentration is increased. When buffer ions are present, they inhibit the transport of the drug by competing with it [16]. Iontophoresis effects vary depending on the tissue on which the electrodes are applied and also vary depending on the thickness, the presence of pores, and permeability. The voltage supply is of two types: alternating current (AC) and direct current (DC). DC iontophoresis is the most commonly used method. According to Zhu et al., in transdermal iontophoresis, better results were shown by AC iontophoresis than conventional constant current (DC) iontophoresis. The probe permeant that they used in this study was mannitol. They found that on a different mechanistic level, constant current DC shows variation in parameters such as pore size, porosity, and surface charge density, while constant current AC iontophoresis effectively maintains the membrane parameters which provide comparatively constant permanent flux [7,17].

Iontophoresis application

With an external electric field, iontophoresis can facilitate the penetration of non-ionic and ionic drugs into the enamel and dentin. Reduction in the incidence of caries and hypersensitivity was observed with iontophoresis treatment. Employing an electric current for drug delivery was introduced several years ago in clinical practice, research, and literature. For treating oral cavity diseases, iontophoresis could improve drug absorption into the enamel, dentin, and other oral tissues. For treatment of tooth decalcification and hypersensitivity and for production of local anesthesia, iontophoresis was assessed in dentistry. This technique evaluated lidocaine and fluoride, which are the most common drugs [15]. There are studies that have investigated the effect of some drugs through iontophoresis on the enamel and dentin, which are discussed below.

Non-Steroidal Anti-Inflammatory Drugs

The delivery of the drug by iontophoresis in unaffected dentin and dentin affected with caries was investigated by Puapichartdumrong et al. Dentinal discs were obtained for the evaluation of drug penetration through iontophoresis and measured hydraulic conductance. Salicylate, naproxen, and metronidazole were used with iontophoresis. It was observed that iontophoresis offers increased transfer of drugs through caries-free and affected dentin. The scanning electron microscopy (SEM) observation of intact dentin showed dentinal tubules left open, while caries-affected dentin showed irregular deposition of minerals. Drug concentrations were determined using a spectrophotometer. The penetration of sodium salicylate, naproxen sodium, and metronidazole was lower through carious dentin than those without caries. The ionized drug showed more penetration through iontophoresis [18].

Local Anesthetics

There is a reversible loss of pain sensation when local anesthetic drugs are used. Anesthetizing effect of lignocaine and epinephrine through iontophoresis was studied by Smitayothin et al. on dentin for cavity preparation. In this study, teeth cavities were filled with lidocaine 20% and epinephrine 0.1%. At 200 mA, an iontophoretic anodal was applied for two minutes. The result showed that iontophoretic transport of lignocaine and epinephrine anesthetized 87.5% of the teeth with caries. There was immediate pain relief after lignocaine with epinephrine solution iontophoresis, which continued for at least 40 minutes. It was also observed that by utilizing AC, the anesthetic lidocaine hydrochloride could pass through the tooth structure. This procedure may help avoid numbness, mostly observed during syringe application [19].

Antibacterial Drugs

Removal of biofilm from root canals of teeth can be done with the help of disinfection by iontophoresis. For the biofilm of gram-positive cocci, iontophoresis with potassium iodide was found to be effective. Cupral (Cu) iontophoresis also had a good bactericidal effect [20]. Silver has an anti-bacterial property. When silver was administered through iontophoresis, it showed a greater anti-bacterial effect against Gram-negative bacteria and lesser against Gram-positive bacteria [21]. A study on radioiodide was conducted to determine the penetration rate of iodide through the enamel surface while using potentials between 1 and 3 volts and its progression towards the pulp, which was compared with the natural diffusion. In this study, when comparing water extracts and acid extracts, it was observed that acid extracts play an important role in the diffusion of drugs through the enamel. Iodide loosely bonded on or near the enamel surface observed with water extracts and acid extracts showed firmly bound iodide or iodide with greater penetration depth. As the tooth carries a negative charge, there is a decrease in anions' entrance rate, such as fluoride and iodide, compared to cations. Therefore, a positive potential was found to increase the penetration rate of radioiodide [22].

Application of iontophoresis in remineralization

The effect of iontophoresis on remineralization and fluoride uptake ability of teeth was evaluated in some studies. Literature showed that fluoride uptake was more with the iontophoresis group than with the conventional application method observed in the following studies. Tanaka et al. compared the iontophoresis method with the immersion method. In this study, fluoride uptake by enamel was measured in both the presence and absence of iontophoresis; 2% fluoride solution was applied to bovine incisors. The fluoride and calcium concentrations were measured with the help of a fluorine ion meter and atomic absorption spectrophotometry, respectively. The fluoride uptake after 3, 5, and 10 minutes were found to be 1.6, 1.9, and 1.5 fold, respectively, by the iontophoresis method. In the immersion method, between 5 and 15 minutes of decalcification time, no changes in the fluoride uptake were observed. A shallower decalcification depth with the iontophoresis was seen. There was a limit on the fluoride uptake through the enamel surface that is only to a depth of 0.12 mm from the enamel surface when fluoride was incorporated [23]. The conventional fluoride application ([CFA] tray technique) and fluoride iontophoresis (FI) method of application of acidulated phosphate fluoride (APF) gel group and sodium fluoride (NaF) varnish group were compared by Kim et al. Demineralized specimens were treated with fluoride for 4 minutes in all experimental groups. For application, NaF (2%) solution and APF (1.23%) gel in the experimental groups were used. The remineralization was checked by measuring lesion depth. Confocal laser scanning microscope (CLSM) imaging was used to measure lesion depth. There was no significant difference between the FI and CFA groups. No superior effect by the iontophoresis group was observed. However, the FI group showed the best result when APF gel (1.23%) was applied with iontophoresis. CLSM analysis showed reduced lesion depth when the 1.23% APF gel was used with iontophoresis compared to the non-varnished group [24]. In another study by Kim et al., the iontophoresis group was compared with the CFA group. All the bovine incisor specimens were treated with fluoride for 4 minutes daily with the help of iontophoresis at different current intensities, such as 100, 200, 300, and 400 μA in the FI group. The concentration of fluoride soluble in potassium hydroxide (KOH) was calculated to assess the quantity of calcium fluoride (CaF2) produced on the tooth surface. No significant difference in the reduction of lesion depth was observed when using microscopy with polarized light. The FI group showed the only increase in fluoride deposition on the enamel surface. In the FI group of 300 μA, the concentration of KOH-soluble fluoride was the highest [25].

For the evaluation of the remineralization effects on initial caries lesion in the enamel, three topical fluoride regimens such as APF (1.23%) gel application, NaF varnish (5%) application, and NaF (2%) solution with iontophoresis were compared. In this study, fluoride uptake, surface hardness, and fluorescence lesion region were observed with the help of a CLSM and digital microhardness tester. Lower lesion depth and higher fluoride uptake were observed in the NaF with iontophoresis group than in the control group. The result also showed that the remineralization effect of NaF iontophoresis was significantly lower than that of APF gel [26]. Pauli et al. evaluated fluoride uptake by applying different current intensities through iontophoresis on dental enamel with artificial carious lesions. The dental block and Ag/AgCl electrodes create a circuit that treats the iontophoresis group for 4 minutes. The fluoride application using 0.8 mA current in the iontophoresis group was compared with the no current application group and the current application of 0.1 mA in the fluoride group. It was discovered that a group treated with fluoride and a current of 0.8 mA produces more fluoro-hydroxyapatite. Therefore, it was confirmed that iontophoresis with more current, that is, 0.8 mA, when combined with the application of fluoridated gel (2% NaF), resulted in further fluoride uptake by caries lesions to form fluorapatite [27]. It was observed that the iontophoretic device was unsuccessful in producing high fluoride precipitation because the flow of fluoride ions is resisted due to polarization generated by the body [28]. Then, for the effective application of fluoride, an iontophoretic device named Fluorinex® was introduced. It aims to increase the tooth's electrical conductivity by pretreatment rinse with CuCl_2_ (copper (II) chloride) solution. Fluoritray® (Fluorinex Ltd, Nazareth, Israel) utilizes low direct electrical current, which leads to the diffusion of fluoride gel into the teeth and the removal of the polarization effect. This results in the active replacement of the hydroxyl group with the fluoride ions. A remineralization effect of the novel iontophoresis device Flurinex (Fluorinex Ltd,) was compared with conventional iontophoresis device and conventional APF gel treatment by measuring with a laser fluorescence device. It was concluded that fluoride application by Fluorinex ensured a superior effect on remineralization as compared to conventional APF gel application and NaF iontophoresis on incipient carious lesions [29].

Application of iontophoresis in hypersensitivity

There are several hypotheses on iontophoresis which state that iontophoresis causes dentin desensitization. Lefkowitz in 1963 proposed a mechanism that states that the reparative dentin is formed due to current application, resulting in dead tract development in primary dentin. Another hypothesis for iontophoresis in modification of the pain-conduction sensory system is that it produces paresthesia through the utilization of electrical current. A third possible explanation is that iontophoresis utilizes electric current, thereby causing the movement of ions that results in the uptake of ions by dentinal tubules and leads to dentinal desensitization [29]. Kaur assessed the effectiveness of three desensitizing agents when applied through iontophoresis. Corticosteroid (methylprednisolone) was compared with 2% NaF, 10% strontium chloride, and distilled water. It was observed that corticosteroids enhance peri-tubular mineralization when applied to dentin. This occlusion of dentinal tubules was seen with the help of SEM analysis. According to SEM analyses, the NaF group had the greatest amount of tubule blockage. This indicates that there would be decreased lumen diameter, reducing fluid movement within dentinal tubules and thereby reducing the sensitivity of dentin [30]. Gangarosa and Park compared the FI with the topical application of fluoride. An impression tray with alginate was used, and 2% NaF was used and directed into the tray. Teeth were treated with this tray; time was set for 10 minutes, and the current was applied between 1 and 1.5 mA. There was a noteworthy decrease in sensitivity by applying iontophoresis with NaF (2%) and an iontophoresis device that they developed. It was concluded that both electrical current and fluoride are required to effectively treat tooth hypersensitivity and that FI is superior to the topical application [31].

In another study class, one cavity was prepared. The iontophoretic application of 2% NaF and desensitization (Parkell Electronic Division, New York, USA) was used. The applicator tip was positioned on the pellet, which was kept in the cavity. Compared to teeth treated with adhesive liner or varnish, those treated with 2% NaF iontophoresis significantly reduced dentinal sensitivity much sooner. Fluoride is applied to the cavity walls and base during NaF iontophoresis, which is predicted to have a cariostatic effect and protect against marginal and recurrent caries. Regarding efficacy, 2% NaF iontophoresis was also found to be most effective in minimizing sensitivity, followed by varnish and SBMP (ScotchBond Multipurpose) [32]. When exposed dentinal lesions were treated with FI, it led to a decrease in sensitivity. FI helps reduce the sensitivity of exposed dentinal tubules, which was analyzed by Carlo et al. This treatment led to a reduction in sensitivity caused by an explorer touch and a blast of air in approximately 90% of patients. Therefore, it was concluded that FI is capable of desensitization. Two methods were compared, including dentin-bonding agent application and acidulated phosphate gel iontophoresis, by Aparna et al., who concluded that the iontophoresis group had effectively reduced hypersensitivity and had fewer failures when compared with dentin-bonding agent application [33,34]. Dentinal hypersensitivity was found to decrease immediately after the use of the iontophoresis technique. This technique was observed to be effective and safe in treating hypersensitivity of dentin.

Application of silver nanoparticles through iontophoresis in dentistry

In dentistry, silver compounds have been used for more than a century, and it was found to have medicinal properties. Silver fluoride (AGF), silver nitrate (AgNO3), silver diamine fluoride (SDF), and other silver particle compounds have been examined and used for the management of caries. It was observed that the use of nanoparticles of silver does not cause discoloration or blackening of teeth. Various studies have explained the cariostatic and remineralizing properties of nanoparticles of silver alone and in combination with many components. As the size of the silver particles decreases, there is an upsurge in the contact surface, which tends to increase the antimicrobial effect and could avoid discoloration or black staining of teeth when SDF is applied. Nanoparticles of silver have a tendency to anchor and diffuse into the bacterial cell wall and cause structural changes in the cell membrane, thereby altering the penetrability of the cell membrane and cell death [35]. A concept of increasing diffusion of silver nanocomposite (AgNC) materials into dentinal tubules by using iontophoresis to target the bacterial source was given by Schwass and Meledandri. Iontophoresis method was utilized for increasing antimicrobial activity by applying electric current on a tooth, thereby driving charged silver nanoparticle (AgNP) assembly into the deep dentinal tubule structures. They proved that penetration of AgNCs into the dentinal tubules after applying an electric field was 10 times deeper than the unassisted driving of ions. The significantly improved antibacterial activity by AgNC assemblies against both Gram-negative bacteria and Gram-positive was seen after the application of DC, thus giving a collaborative current effect or antibacterial effect on the survival of all the species of microorganisms tested [36]. Therefore, iontophoresis could be an effective method for transferring remineralizing material, and a reduction in the size of remineralizing material may result in deeper penetration. There is a chance that they will diffuse into the subsurface lesion to promote demineralization. Table 1 summarizes the iontophoresis studies (on enamel and dentin) reviewed in the present paper.

**Table 1:** Overview of the studies involving the effect of iontophoresis on enamel and dentin surface CLSM, confocal laser scanning microscope; NaF, sodium fluoride; VAS, visual analog scale; VRS, verbal rating scale

Author	Criteria	Interventions	Analysis	Results
Gangarosa and Park (1978) [31]	Hypersensitivity	Voltage: 40 V DC duration: 10 minutes Current: 1 to 1.5 mA. (Each tooth received one mA/min of electricity. The operating mA is set to a maximum of 2 mA so that the patient’s threshold of sensation is never exceeded.)	VAS	Fluoride iontophoresis in cavity preparation, enamel hypoplasia, and cement restoration. Decrease in sensitivity with the use of iontophoresis with 2% NaF.
Smitayothin et al. (2015) [19]	Anesthetizing effect of lignocaine and epinephrine	Current: 200 μA. Duration: 2 minutes	VAS	Immediate relief of pain after lignocaine with epinephrine solution iontophoresis which continued for at least 40 min
Kim et al. (2009) [24]	Lesion depth, Remineralization	Current: 0.4 mA Voltage: 12 V Duration: 4 min	Vickers surface microhardness, CLSM	The NaF varnish group's remineralization effect was not superior to that of the fluoride iontophoresis group. Reduced lesion depth when the 1.23% APF gel was used with iontophoresis.
Kim et al. (2016) [25]	Lesion depth, Remineralization	Fixed voltage of 12V. Current: 100 µA to 400 µA. Duration: 4 minutes	Polarized light microscopy	No significant difference in lesion depth reduction, increase in the concentration of fluoride by iontophoresis
Stowell et al. (1964) [22]	Iodide uptake	1 volt and 3 volts	Radioautographic study	An increase in the penetration of iodide into enamel was seen. A highly significant acceleration can be produced with a voltage of as little as 1 volt.
Puapichartdumrong et al. (2003) [18]	Uptake of drugs (salicylate, naproxen, and metronidazole)	Current: 0.05 mA. Duration: 10 minutes	Spectrophotometer, scanning electron microscopy	Increase transfer of drugs through caries-free and affected dentin
Gergova et al. (2016) [20]	Antibacterial Drugs for disinfecting root canals of teeth	Current: 1.5 mA electric current. Duration: 10 minutes	Scanning electron microscopy	For the biofilm of gram-positive cocci, iontophoresis with potassium iodide was found to be effective. Cupral (Cu) iontophoresis also had a good bactericidal effect.
Tanaka et al. (2018) [23]	Fluoride uptake	Current: 200, 400, and 500 µA. Duration: 3 and 5 minutes	A fluorine ion meter was used to test the concentration of fluoride, and atomic absorption spectrophotometry was utilized to assess the calcium concentration.	Fluoride uptake could be increased. Decalcification caused by acid could be reduced.
Lee et al. (2010) [26]	Fluoride uptake, lesion depth, and remineralization	Current: 200 µA. Duration: 4 minutes	Digital microhardness tester, CLSM	Lower lesion depth and higher fluoride uptake were observed in the iontophoresis NaF group than in the control group. Naf iontophoresis has a substantially less remineralization effect than APF gel.
Pauli et al. (2019) [27]	Uptake of fluoride by applying different current intensities	Current: 0.1, 0.2, 0.4, and 0.8 mA. Duration: 4 minutes	Knoop microhardness tester	Increased electric current results in increased deposition of fluoride
Girenes and Ulusu (2014) [29]	Remineralization effect	Current: 200 mA. Duration: 4 minutes	Laser fluorescence device	Compared to conventional APF gel application and NaF iontophoresis, Fluorinex assured better demineralization on incipient carious lesions
Kaur (2016) [30]	Dentin hypersensitivity	Current: 0.5 mA. Duration: 2 minutes	VRS and VAS	Corticosteroids Iontophoresis enhances peri-tubular mineralization when applied to dentin
Schwass and Meledandri (2014) [36]	Diffusion of silver nanocomposite materials into dentinal tubules	Current: 0.12 mA	Electrical conductivity, dynamic light scattering, scanning and transmission electron microscopy, and inductively coupled plasma mass spectrometry were used to identify silver nanoparticles. Crystal violet assay was used to calculate the mass of the biofilm.	Penetration of silver nanocomposites into the dentinal tubules after application of an electric field was 10 times deeper than the unassisted driving of ions.

Discussion

Iontophoretic drug delivery is considered non-invasive and safe. Developing an effective iontophoresis method for local drug administration is significant since the currently available dental local drug delivery techniques are not particularly practical and efficient. Although iontophoresis has been employed in dental and oral care, this technology has not yet been completely utilized [15]. Iontophoresis may be a successful drug delivery method for treating oral conditions such as hypersensitivity and tooth decalcification. As this technique facilitates accelerated movement of ions while being affected by an external electrical potential difference, it may cause deeper penetration of drugs into the teeth. The mechanism of iontophoresis explains that drug-carrying charges play an important role. One of the studies concluded that when NaF was used, anions of fluoride formed. As fluoride is highly electronegative, they enter into the dentinal tubules when used with iontophoresis compared to the topical application, which is only deposited on the surface [15]. The type of current used also seems to play an important role in effective drug delivery. It was found that AC shows a greater transfer of current than DC. Also, a study showed that anesthetic drugs and DC/AC iontophoresis resulted in greater drug delivery [17]. The teeth's positive and negative potential also decides the drug's entry. If ions with a negative charge are needed to be transferred into the tooth structure, then the tooth needs to carry a positive charge. Therefore, one of the studies found that when a tooth is treated with acid, there is more drug penetration [29]. The iontophoresis method helps in remineralization. Fluoride uptake by enamel was found to be greatly increased by iontophoresis. With the increase in time, the drug was more precipitation compared to the immersion method. The concentration of fluoride and calcium was measured with the help of a fluorine ion meter and atomic absorption spectrophotometry, respectively [23]. A study by Kim et al. found reduced lesion depth in the FI group when analyzed with a CLSM. But this result was not superior to the conventional application method. The limitation of this study is that there could be histologic differences in the area of application of teeth and skin, electrochemical properties of FI were not considered, and the FI setting mode was established following the directions in the product manual [24]. Therefore, in another study by Kim et al., the effect of iontophoresis was evaluated using different current intensities. The concentration of fluoride soluble in KOH was calculated to assess the quantity of CaF2 produced on the tooth surface. It was observed that with more current, there was CaF2 deposition on the tooth surface. Also, they found that remineralization can occur regardless of the treatment with a high fluoride concentration through iontophoresis [25].

When different regimens effects were analyzed, it was seen that APF showed a greater remineralization effect as compared to the NaF iontophoresis group. This can be understood by the fact that acid in the APF gel plays a major role. However, in this study, only microhardness testing was used to determine the quantitative analysis of the lesion depth. The observation time was also limited. Therefore, this study does not demonstrate the effects of topical fluoride when used over the long term [24,26]. There was some advancement in the iontophoretic devices because of polarization generated by the body and there was resistance offered to the fluoride precipitation. Therefore, Fluorinex, a Fluoritray was invented, which utilizes low current and removes the polarization effect. It showed a superior remineralization effect [29]. When two methods were compared for the evaluation of hypersensitivity, including dentin-bonding agent application and acidulated phosphate gel iontophoresis, the iontophoresis group had less failure [34]. Iontophoresis offered a reduction in hypersensitivity by occlusion of dentinal tubules. Most in vitro studies observed these findings with the help of SEM analysis [18,20]. In vivo studies involved different rating scales, including the visual analog scale and a verbal rating scale [19,24,31]. The Iontophoresis technique effectively reduces hypersensitivity because of the greater penetration rate of ions into the dentinal tubules offered by electric current. There are limited studies about the mechanisms of iontophoretic transport for the oral mucosa, enamel, and dentin, despite studies showing improved ions and drug penetration through iontophoresis for local administration. Many studies showed increased precipitation and surface deposition of drugs through iontophoresis but no subsurface deposition into the tooth structure [18,22,24-27,36]. To increase its efficacy, drugs with smaller particle sizes may be infused. Because of this, there is a chance that the nanocomplexes could penetrate the body of the subsurface lesion through the porosities. A study conducted by Schwass and Meledandri included AgNC materials through iontophoresis. It was observed that ions were more penetration into the dentinal tubules with smaller particle sizes. Also, there was a significantly increased anti-bacterial effect. Iontophoresis using nanoparticles may therefore be beneficial for subsurface lesions [36].

## Conclusions

In iontophoresis, effective drug delivery was seen when drugs with ionic charges were used. A tooth with positive and negative potential plays an essential role in the penetration of drug. With the increase in current intensity, there was an increase in the deposition of the drug. Iontophoresis dramatically enhances both the rate of release and the extent of penetration of the drugs. Most studies showed surface deposition of ions; therefore, to improve subsurface demineralization, drugs with smaller particle sizes should be used. Nanomaterials can be used with iontophoresis. Therefore, studies on iontophoresis with nanomaterials need to be conducted.

## References

[1] Goldberg M, Arends J, Septier D, Jongebloed WL (1981). Microchannels in the surface zone of artificially produced caries-like enamel lesions. J Biol Buccale.

[2] Aas JA, Griffen AL, Dardis SR (2008). Bacteria of dental caries in primary and permanent teeth in children and young adults. J Clin Microbiol.

[3] Schwendicke F, Walsh T, Fontana M (2018). Interventions for treating cavitated or dentine carious lesions. Cochrane Database Syst Rev.

[4] Khan A, Yasir M, Asif M (2011). Iontophoretic drug delivery: history and applications. Jr Appl Pharm Sci.

[5] Suchetha A, Rajeshwari HR, Mundinamane DB (2015). Novamin versus sodium flouride iontophoresis: the “salvage crew” to the rescue. Int Res J Medical Sci.

[6] Patil AR, Varma S, Suragimath G, Abbayya K, Zope SA, Kale V (2017). Comparative evaluation of efficacy of iontophoresis with 0.33% sodium fluoride gel and Diode laser alone on occlusion of dentinal tubules. J Clin Diagn Res.

[7] Karpiński TM (2018). Selected medicines used in iontophoresis. Pharmaceutics.

[8] Dhote V, Bhatnagar P, Mishra PK, Mahajan SC, Mishra DK (2012). Iontophoresis: a potential emergence of a transdermal drug delivery system. Sci Pharm.

[9] Arowojolu MO (2002). Fluoride iontophoresis versus topical fluoride application in the treatment of dentine hypersensitivity. Niger J Clin Pract.

[10] Kalia YN, Naik A, Garrison J, Guy RH (2004). Iontophoretic drug delivery. Adv Drug Deliv Rev.

[11] Pikal MJ (2001). The role of electroosmotic flow in transdermal iontophoresis. Adv Drug Deliv Rev.

[12] Costello CT, Jeske AH (1995). Iontophoresis: applications in transdermal medication delivery. Phys Ther.

[13] Becker SM, Kuznetsov AV (2022). Transport In Biological Media.

[14] Ren W, Baig A, Li SK (2014). Passive and iontophoretic transport of fluorides across enamel in vitro. J Pharm Sci.

[15] Wanasathop A, Li SK (2018). Iontophoretic drug delivery in the oral cavity. Pharmaceutics.

[16] Rai R, Srinivas CR (2005). Iontophoresis in dermatology. Indian J Dermatol Venereol Leprol.

[17] Zhu H, Li SK, Peck KD, Miller DJ, Higuchi WI (2002). Improvement on conventional constant current DC iontophoresis: a study using constant conductance AC iontophoresis. J Control Release Off J Control Release Soc.

[18] Puapichartdumrong P, Ikeda H, Suda H (2003). Facilitation of iontophoretic drug delivery through intact and caries-affected dentine. Int Endod J.

[19] Smitayothin TL, Vongsavan K, Rirattanapong P, Kraivaphan P, Vongsavan N, Matthews B (2015). The iontophoresis of lignocaine with epinephrine into carious dentine for pain control during cavity preparation in human molars. Arch Oral Biol.

[20] Gergova RT, Gueorgieva T, Dencheva-Garova MS, Krasteva-Panova AZ, Kalchinov V, Mitov I, Kamenoff J (2016). Antimicrobial activity of different disinfection methods against biofilms in root canals. J Investig Clin Dent.

[21] Shirwaiker RA, Wysk RA, Kariyawasam S, Voigt RC, Carrion H, Nembhard HB (2014). Interdigitated silver-polymer-based antibacterial surface system activated by oligodynamic iontophoresis - an empirical characterization study. Biomed Microdevices.

[22] ST EC, TA JB (1964). Influence of electrical potential on ion migration in teeth. 2. Quantitative measurements of I-131 penetration by an acid-leaching technique. J Dent Res.

[23] Tanaka T, Saito A, Watanabe K, Saeki K, Nakashima H, Maki K (2018). Preventive effects of iontophoresis on bovine enamel decalcification through enhancing uptake and transportation of fluoride - in vitro study. Pediatr Dent J.

[24] Kim HE, Kwon HK, Kim BI (2009). Application of fluoride iontophoresis to improve remineralization. J Oral Rehabil.

[25] Kim HE, Kim BI (2016). Can the application of fluoride iontophoresis improve remineralisation of early caries lesions?. Oral Health Prev Dent.

[26] Lee YE, Baek HJ, Choi YH, Jeong SH, Park YD, Song KB (2010). Comparison of remineralization effect of three topical fluoride regimens on enamel initial carious lesions. J Dent.

[27] Pauli MC, Tabchoury CP, Silva SA, Ambrosano GM, Lopez RF, Leonardi GR (2019). Effect of iontophoresis on fluoride uptake in enamel with artificial caries lesion. Braz Oral Res.

[28] Gangarosa LP Sr (1981). Iontophoretic application of fluoride by tray techniques for desensitization of multiple teeth. J Am Dent Assoc.

[29] Girenes G, Ulusu T (2014). An in vitro evaluation of the efficacy of a novel iontophoresis fluoride tray on remineralization. J Clin Exp Dent.

[30] Kaur DM (2016). Methyl prednisolone with iontophoresis in the treatment of dentine hypersensitivity. An In-vitro and in-vivo study. IOSR Jr Dent Med Sci.

[31] Gangarosa LP, Park NH (1978). Practical considerations in iontophoresis of fluoride for desensitizing dentin. J Prosthet Dent.

[32] Gupta M, Pandit IK, Srivastava N, Gugnani N (2010). Comparative evaluation of 2% sodium fluoride iontophoresis and other cavity liners beneath silver amalgam restorations. J Indian Soc Pedod Prev Dent.

[33] Butrón Téllez Girón C, Hernández Sierra JF, DeAlba-Montero I, Urbano Peña ML, Ruiz F (2020). Therapeutic use of silver nanoparticles in the prevention and arrest of dental caries. Bioinorg Chem Appl.

[34] Aparna S, Setty S, Thakur S (2010). Comparative efficacy of two treatment modalities for dentinal hypersensitivity: a clinical trial. Indian J Dent Res.

[35] Sondi I, Salopek-Sondi B (2004). Silver nanoparticles as antimicrobial agent: a case study on E. coli as a model for Gram-negative bacteria. J Colloid Interface Sci.

[36] Schwass DR, Meledandri CJ (2022). Enhanced penetration of silver nanocomposite assemblies into dentine using iontophoresis: toward the treatment of dental caries. Chem Plus Chem.

